# 

**Published:** 1850-04

**Authors:** 


### CONTENTS

### OF NO. IV.

### ORIGINAL COMMUNICATIONS.

### ESSAYS AND CASES.

ART. I.—Two Lectures on Water. Delivered to his Class, in Lebanon, Tennessee, by F. H. GORDON, M. D - 277

ART. II.—Case of Malformation of the Uterus, with Fatal Post-Partum Hemorrhage; Morbid Adhesion, and Partial Osseous Degeneration of the Placenta. By R. C. HEWETT, M. D., of Louisville, Kentucky. - - - 300

ART. III.—A Case of Chronic Pneumonia, with Anomalous Physical Signs—Abscess.—Death.—Autopsy. By D. W. YANDELL, M. D., of Louisville, Ky., Attending Physician for the Faculty at the Louisville Marine Hospital. - - - - - - 312

ART. IV.—A Case of Traumatic Tetanus.—Recovery. By SAMUEL S. EMISON, of Scott county, Ky. - - 317

ART. V.—Remarkable Complication of Diseases. By CLAIBORNE PIRTLE, M. D., of Louisville, Ky. - - 320

### REVIEWS.

ART. VI.—On the Treatment of Gun-Shot Wounds. By BENJ. W. DUDLEY, M. D., Professor of Surgery in Transylvania University. Transylvania Medical Journal, Vol. i., No. 3. Dec., 1849.

On the Use of the Bandage in Gun-Shot Wounds, etc. By BENJAMIN WINSLOW DUDLEY, M. D., Professor of Anatomy and Surgery in the Transylvania University. Transylvania Journal of Medicine, etc., Vol. i., p. 501. 1828. - - - - - - 324

ART. VII.—A Theoretical and Practical Treatise on Midwifery, including the Diseases of Pregnancy and Parturition By P. Cazeaux, Adjunct Prof. in the Faculty of Medicine of Paris, etc., etc. (Adopted by the Royal Council of Public Instruction.) Translated from the second French edition, with occasional notes and a copious index, by Robert P. Thomas, M. D. - - - 359

Art. VIII.—The Annual of Scientific Discovery; or, Year Book of Facts in Science and Art—Exhibiting the most important discoveries and improvements in mechanics, useful arts, natural philosophy, chemistry, astronomy, meteorology, zoology, botany, mineralogy, geology, geography, antiquities. Together with a list of recent scientific publications; a classified list of patents; obituaries of eminent scientific men; an index of important papers in scientific journals, reports, etc. Edited by David Wells, of the Lawrence Scientific School, Cambridge, and George Bliss, Jr. - - - - 365

ORIGINAL INTELLIGENCE.

Postponed Items, - - - - - - - 366
The Position of the Medical Profession in Society, - 366
Sanitary Reform, - - - - 367

